# A voice recognition-based digital cognitive screener for dementia detection in the community: Development and validation study

**DOI:** 10.3389/fpsyt.2022.899729

**Published:** 2022-07-22

**Authors:** Xuhao Zhao, Ruofei Hu, Haoxuan Wen, Guohai Xu, Ting Pang, Xindi He, Yaping Zhang, Ji Zhang, Christopher Chen, Xifeng Wu, Xin Xu

**Affiliations:** ^1^School of Public Health and The Second Affiliated Hospital of School of Medicine, Zhejiang University, Hangzhou, China; ^2^Life Support Technologies Group, Technical University of Madrid, Madrid, Spain; ^3^DAMO Academy, Alibaba Group, Hangzhou, China; ^4^Memory, Ageing, and Cognition Centre (MACC), Department of Pharmacology, Yong Loo Lin School of Medicine, National University of Singapore, Singapore, Singapore

**Keywords:** digital cognitive screening, dementia, MCI, MoCA, validity, reliability

## Abstract

**Introduction:**

To facilitate community-based dementia screening, we developed a voice recognition-based digital cognitive screener (digital cognitive screener, DCS). This proof-of-concept study aimed to investigate the reliability, validity as well as the feasibility of the DCS among community-dwelling older adults in China.

**Methods:**

Eligible participants completed demographic, clinical, and the DCS. Diagnosis of mild cognitive impairment (MCI) and dementia was made based on the Montreal Cognitive Assessment (MoCA) (MCI: MoCA < 23, dementia: MoCA < 14). Time and venue for test administration were recorded and reported. Internal consistency, test-retest reliability and inter-rater reliability were examined. Receiver operating characteristic (ROC) analyses were conducted to examine the discriminate validity of the DCS in detecting MCI and dementia.

**Results:**

A total of 103 participants completed all investigations and were included in the analysis. Administration time of the DCS was between 5.1–7.3 min. No significant difference (*p* > 0.05) in test scores or administration time was found between 2 assessment settings (polyclinic or community center). The DCS showed good internal consistency (Cronbach’s alpha = 0.73), test-retest reliability (Pearson *r* = 0.69, *p* < 0.001) and inter-rater reliability (ICC = 0.84). Area under the curves (AUCs) of the DCS were 0.95 (0.90, 0.99) and 0.77 (0.67, 086) for dementia and MCI detection, respectively. At the optimal cut-off (7/8), the DCS showed excellent sensitivity (100%) and good specificity (80%) for dementia detection.

**Conclusion:**

The DCS is a feasible, reliable and valid digital dementia screening tool for older adults. The applicability of the DCS in a larger-scale community-based screening stratified by age and education levels warrants further investigation.

## Introduction

Dementia has long become a global public health problem, which will not only seriously damage the quality of life of patients, but also cause a huge social burden (1, 2).

Screening can alert people to early signs of cognitive decline, leading to better allocation of healthcare resources and reduced healthcare cost. Early detection also provides an optimal window of early intervention and treatment which has been proven to slow down cognitive decline and reduce the risk of dementia conversion (3, 4). Therefore, timely identification of potential at-risk older adults is the essential first step to delaying dementia onset, so as to render support to individual older adults, caregivers, healthcare providers, as well as the whole society (5). As a result, researchers have emphasized the necessity of early screening, particularly the implementation of simple and efficient assessment tools in various healthcare settings.

Although traditional paper-pencil tests are commonly applied, most of them must be performed by trained assessors and take a long time for face-to-face administration (6, 7). In contrast, digital cognitive testing provides new opportunities for regular self-accessible assessment and remote monitoring of cognitive changes. Digital cognitive screening overcomes the various obstacles of traditional testing. First of all, it can be carried out with minimum or no assistance of non-professional staff or family members, which makes it more flexible for anytime- and anywhere-assessment, and hence feasible for regular monitoring of cognitive changes (8). This is particularly helpful in when the COVID pandemic has impacted adversely on the healthcare routines in the communities (9). In addition, the analytic platform equipped with the assessment tool allows not only efficient management of assessment data, but also automatic execution of the entire assessment process. As such, digital cognitive screeners are believed to have a promising prospect in facilitating large-scale cognitive screening in the community.

So far, digital cognitive assessments are mostly developed by adapting various cognitive tests (8). However, most of the presently available cognitive tests are touch screen-based, which requires operation from individual participant. This has resulted in lower test acceptance and performance due to computer illiteracy among older adults who are less familiar with operating digital devices (10, 11). Furthermore, even though mobile platforms can collect new data streams and achieve a high measurement accuracy, they are usually expensive to track longitudinal behavioral/cognitive changes, such as sensor data through wearables (12, 13). All the above challenges have been brought up as important obstacles to be overcome.

Therefore, in the present study, we developed a brief digital cognitive tool (digital cognitive screener, DCS) based on a voice-recognition machine-learning system that runs on mobile devices. The DCS simulates a standard face-to-face cognitive test conducted by professional testers. The DCS was adapted from the montreal cognitive assessment (MoCA) which is a well-validated cognitive test and can be conducted verbally through the telephone (14, 15). In this study, we aimed to investigate the reliability and validity as well as the feasibility of the DCS among community-dwelling older adults in China. We hypothesized that the DCS had good validity which can be demonstrated by the diagnostic accuracy by establishing sensitivity, specificity, receiver operating characteristics (ROC), area under the curve values (AUC) and optimal cut-offs.

## Materials and methods

### Study participants

From July to October 2021, 104 participants aged 65 and above were recruited from local primary healthcare facilities (polyclinics) and communities in Hangzhou, China. Participants completed demographic, clinical, and cognitive assessments (MoCA), followed by the DCS after a two-week interval. Exclusion criteria were: (1) significant sensory impairment, e.g., verbal and hearing impairment, etc. and (2) presence of major depression and other psychiatric disorders.

This study was approved by Medical Ethics Committee in Zhejiang University School of Public Health (ZGL202101-1) and written informed consent was obtained from all participants or their legally acceptable representatives.

### Demographic and clinical assessments

Demographics (age, sex, education level), smoking status and medical history of cerebrovascular disease (CVD), heart disease, hypertension, dyslipidemia and diabetes were recorded.

### Cognitive assessments and diagnosis

The MoCA was administrated by trained research personnel. The MoCA is a well-validated multidomain paper-and-pencil cognitive test that assesses visuospatial and executive functions, naming, memory, attention, abstraction, and orientation, with the administration time about 15 min.

It has been validated among Chinese older adults (7). In this study, we defined cognitive status by MoCA cutoff scores: (1) no cognitive impairment (NCI) as MoCA ≥ 23; (2) mild cognitive impairment (MCI) as MoCA < 23 and ≥ 14 (16); and (3) dementia as MoCA < 14 (17).

### Digital cognitive screener administration

The DCS included the following cognitive test items (3, 4): (1) Memory: 5-word (face, silk, chrysanthemum, hotel, red) delayed memory test with a total score of 5; (2) Orientation: 6-item orientation task (year, month, week, date, city, address) with a total score of 6; and (3) Executive function: animal fluency test with a score of 1 if participants speak out more than 11 animals. We applied the DCS among participants recruited from polyclinics and the community to explore if the administration time and test score differ between different settings. Among all participants, 56 took the DCS assessment twice (test interval = 14 days).

The DCS platform is a semi-automatic conversational robot (18) and can be conducted by non-professional facilitators, such as caregivers or social workers. Using voice-recognition technology, the platform initiates a conversation by asking the questions and cuing participants to answer verbally. The facilitator then prompts the system to continue by providing feedback on whether the participant has answered the question. The feedback options displayed on-screen for the facilitators are simple description of the participant’s reaction, such as “the participant didn’t respond” or “the participant responded.” As such, the facilitators do not need to evaluate older adults’ performance themselves.

After the test is finished, the platform automatically scores the participants’ performance based on their verbal response. The automatic speech recognition (ASR) converts the test audio into text, and subsequently, natural language understanding (NLU) is used to extract key information from the text data and assess its correctness. Specifically, a named entity recognition model is implemented to extract the key information such as year, day, and address in the text. After that, the extracted result is compared with the correct standard answer. For each question, the platform automatically analyzes the answer and assigns the score. Finally, the platform adds up all scores to obtain a final test result for each participant. In order to explore whether the automated scoring is consistent with traditional manual scoring, trained research assistants reviewed the test audios and performed a blinded scoring process. Each audio was scored by 3 independent raters.

**FIGURE 1:**
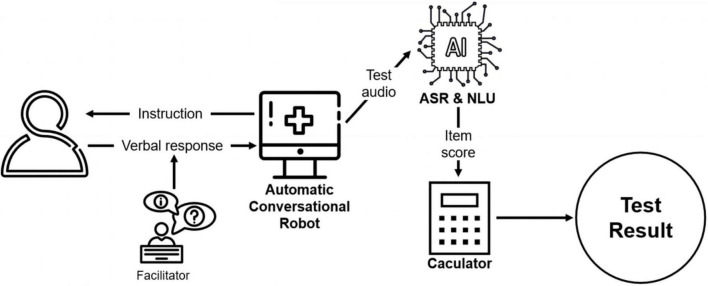
Administration process of DCS.

The administration process of DCS is shown in Figure 1.

### Statistical analysis

Demographic characteristics and cognitive outcomes between the NCI, MCI and dementia groups were compared by one-way ANONA and Chi-square testes for continuous and categorical variables, respectively. *Post hoc* analysis by Fisher’s Least Significant Difference (LSD) method were used for stepwise comparison.

Internal consistency of the DCS was examined with Cronbach’s alpha. Test-retest reliability of the DCS’s total score was measured using Pearson’s correlation analysis. Inter-rater reliability of manual scores was measured by the inter-class correlation coefficient (ICC). Association between the automated and manual scoring was measured by Pearson’s correlation. Agreement between automated and manual scoring was analyzed using the Bland-Altman plot and calculating Cohen’s kappa on categorizing dementia and MCI. To study the effect of age and education on the DCS, a repeated measures ANOVA was implemented to examine the difference between the automated and manual scoring with age and education as indicators.

To examine discriminate validity, the receiver operating characteristic curve was employed to establish the area under the curves (AUCs) of the DCS for different cognitive status. Discriminant indices, including sensitivity, specificity, positive and negative predictive value (PPV and NPV) were calculated using the optimal cut-off points. We categorized the MCI and dementia as cognitive impairment (CI) and NCI and MCI as dementia-free to investigate the validity in different cognitive outcomes. Subgroup analysis was done according to age and education stratifications. All analyses were performed with R, setting statistical significance level at < 0.05.

## Results

### Characteristics of participants

**FIGURE 2:**
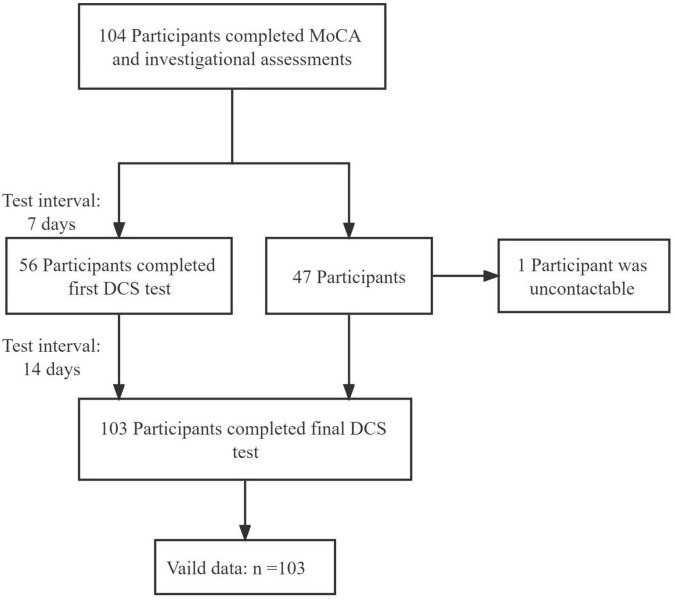
Flow diagram of participants.

**FIGURE 3:**
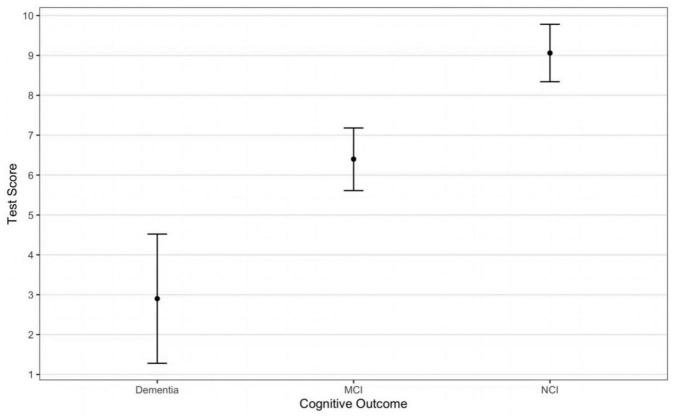
Participants test score on the DCS by cognitive outcomes.

The study flowchart is presented in Figure 2. Only 1 participant was lost to contact. Participant characteristics are shown in Table 1. The DCS performance differed among varying cognitive groups [*F*_(2, 107)_ = 43.21, *p* < 0.001] (Figure 3).

**TABLE 1:** Participant characteristics.

Characteristics	Dementia (*N* = 10)	MCI*^a^* (*N* = 43)	NCI*^b^* (*N* = 50)	Total (*N* = 103)	*P*-value
Age (years, mean ± SD)	71.9 ± 7.5	71.3 ± 6.1	70.4 ± 5.8	70.9 ± 6.0	0.67
Sex (male, %)	1 (10.0%)	12 (27.9%)	13 (26.0%)	25 (24.3%)	0.45
Education (years, mean ± SD)	4.5 ± 5.3	6.8 ± 3.2	9.7 ± 3.4	7.7 ± 3.8	< 0.001
Smoking (%)	1 (10.0%)	4 (13.3%)	4 (12.9%)	8 (11.3%)	0.48
CVD*^c^* (%)	2 (20.0%)	4 (13.3%)	1 (3.2%)	7 (9.9%)	0.21
Heart disease (%)	3 (30.0%)	11 (36.7%)	6 (19.4%)	20 (28.2%)	0.32
Hypertension (%)	2 (20.0%)	15 (50.0%)	14 (45.2%)	31 (43.7%)	0.25
Dyslipidemia (%)	1 (10.0%)	12 (40.0%)	11 (35.5%)	24 (33.8%)	0.21
Diabetes (%)	1 (10.0%)	3 (10.0%)	5 (16.1%)	9 (12.7%)	0.74
AD8-slef*^d^* (points, mean ± SD)	2.1 ± 1.8	1.8 ± 1.9	1.5 ± 1.6	1.6 ± 1.7	0.82
DCS*^e^* (score, mean ± SD)	2.9 ± 2.3	6.4 ± 2.8	9.1 ± 2.4	7.4 ± 3.2	< 0.001
MoCA*^f^* (points, mean ± SD)	9.90 ± 1.97	18.6 ± 2.72	25.7 ± 2.04	21.2 ± 5.5	< 0.001

^a^MCI, mild cognitive impairment; ^b^NCI, no cognitive impairment; ^c^CVD, cerebrovascular disease; ^d^AD8-info, Ascertain Dementia 8- self version; ^e^DCS, digital cognitive screener; ^f^MoCA, Montreal Cognitive Assessment.

### Feasibility

**FIGURE 4:**
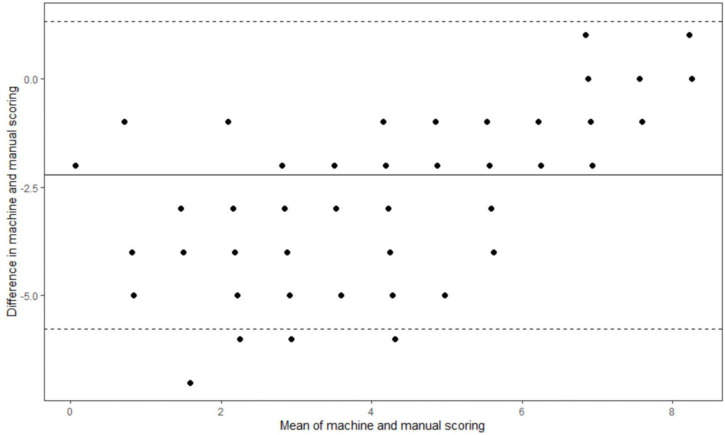
Bland-Altman plot of machine and manual scoring.

The average administration time of the DCS was between 5.1–7.3 min. The automated scores were significantly associated with those scored by human examiners (manual scores) (*r* = 0.83, *p* < 0.001) yet were on average 2 point lower than scores by manual evaluation (automated score vs. manual score: 7.4 ± 3.2 vs. 9.5 ± 2.4, *p* < 0.05). Results from the repeated measures ANOVA after controlling for age and education showed that the difference between the automated and manual scoring was comparable to the results without adjustment (estimated difference = 2.1, *F* = 139.44, *p* < 0.05). No significant difference (*p* > 0.05) was found in test scores and administration time when conducting the DCS at two different settings (polyclinics and community). The Cohen’s Kappa between automated and manual scoring on dementia and MCI were 0.62 and 0.73, respectively. The Bland-Altman plot is shown in Figure 4.

### Reliability

Internal consistency of the DCS was shown by the Cronbach’s alpha of 0.84 for manual scoring and 0.82 for automated scoring. The overall test-retest reliability among the 56 participants who were tested twice (test interval ≥ 7 days) was represented by the Pearson correlation of 0.69 (*p* < 0.001) and 0.71 (*p* < 0.001) for manual and automated scoring, respectively. The inter-rater reliability analysis showed the ICC of the overall test score was 0.84.

### Validity

**FIGURE 5:**
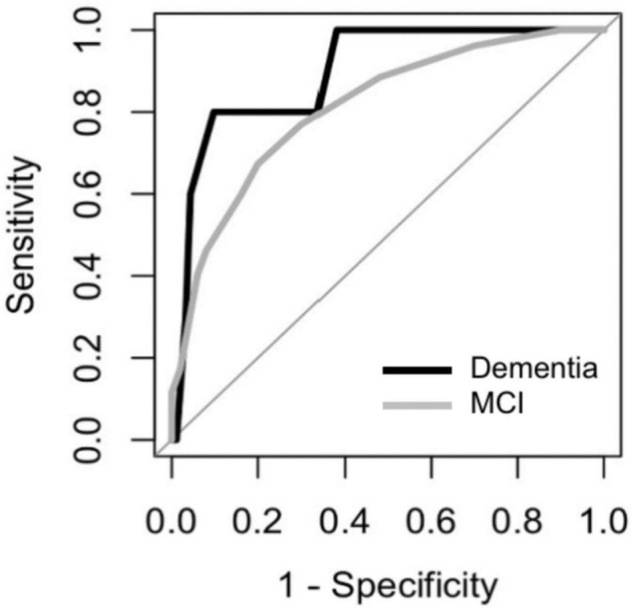
Receiver Operating Characteristic (ROC) curves of the DCS for discriminating among participants with MCI and dementia.

The ROCs of the DCS for dementia and MCI were shown in Figure 5. The discriminate incidences of the DCS on different cognitive outcomes are shown in Table 2 (both automated and manual scoring results are shown). No significantly difference (all *p*s > 0.05) of AUC was found between the manual and automated scoring for each cognitive outcomes, demonstrating an equivalent discriminating ability between these two scoring approaches.

**TABLE 2:** Diagnostic ability of the DCS on different cognitive outcomes.

Cognitive outcomes	Cut-off	AUC (95% CI)	Sensitivity	Specificity	PPV*^b^*	NPV*^c^*
**NCI*^a^* vs. Dementia**						
Automated scoring	7/8	0.95 (0.90,0.99)	100%	80%	50%	100%
Manual scoring	7/8	0.99 (0.98,1.00)	80.0%	100%	100%	96.2%
**Dementia free vs. Dementia**						
Automated scoring	7/8	0.89 (0.81,0.98)	100%	62.4%	22.2%	100%
Manual scoring	7/8	0.90 (0.87,0.99)	80.0%	89.2%	44.4%	97.6%
**NCI vs. MCI*^d^***						
Automated scoring	8/9	0.77 (0.67,0.86)	69.8%	70.0%	66.7%	72.9%
Manual scoring	8/9	0.81 (0.73,0.90)	51.2%	92.0%	84.6%	68.7%
**NCI vs. CI*^e^***						
Automated scoring	8/9	0.80 (0.72,0.89)	75.5%	70.0%	72.7%	72.9%
Manual scoring	8/9	0.84 (0.77,0.92)	60.4%	92.0%	88.9%	68.7%

^a^NCI, no cognitive impairment; ^b^PPV, positive predictive value; ^c^NPV, negative predictive value; ^d^MCI, mild cognitive impairment; ^e^CI, cognitive impairment.

Table 3 shows the diagnostic ability of the DCS in different subgroups. The DCS showed better discriminant validity among more educated (education years > 6) and older (older than 75 years) participants for detecting dementia while the diagnostic ability for MCI was best among older participants.

**TABLE 3:** Diagnostic ability of the DCS in different subgroups.

Cognitive outcomes	Cut-off	AUC	Sensitivity	Specificity
**Dementia free vs. Dementia**				
Education years ≤ 6	7/8	0.78	100%	43.3%
Education years > 6	5/6	0.99	100%	92.6%
Age, years < 75	7/8	0.87	100%	64.3%
Age, years ≥ 75	3/4	0.95	100%	86.7%
**NCI*^a^* vs. CI*^b^***				
Education years ≤ 6	8/9	0.73	75.9%	62.5%
Education years > 6	9/10	0.78	72.2%	71.4%
Age, years < 75	8/9	0.77	73.7%	71.1%
Age, years ≥ 75	7/8	0.88	83.3%	80.7%

^a^NCI, no cognitive impairment; ^b^CI, cognitive impairment.

## Discussion

The present study found that the DCS had good reliability, validity and feasibility among Chinese community-dwelling older adults. Furthermore, the tool has excellent discriminating capacity in detecting dementia patients, but a moderate to fair capacity in detecting MCI patient from normal older adults.

We found that the diagnostic ability as well as optimal cut-offs of the DCS differed between age and education levels. This result is accordance with previous evidence which demonstrated that the performance on cognitive tests (including the 5-min MoCA) is affected by these two factors, especially among Asian countries (19, 20).

The need for a smart and digital screening platform has already been heightened to cater for a wide population of at-risk individuals. Adapted from the MoCA, the development of the DCS was in accordance with the main strategy of developing accurate and brief mobile screening among older adults (8). The excellent sensitivity and good specificity enable the DCS to detect dementia cases who need further cognitive assessment and rule out most cognitively healthy older adults. In the future, we are planning to develop a fully automatic AI version of the DCS which can interact with the participant without the operator and that will promote further implementation of our platform to cater for self-help at-home screening. The fully automatic AI version will be implemented by collaborating with local government for non-commercial utility. All information will be anonymous and only be used for cognitive screening purpose by authorized personnel.

Test length, administration mode and the number of test items can notably impact the effectiveness and efficiency of cognitive screening, especially in a larger population (20). It has been recommended that a shorter cognitive test could induce less mental fatigue and encourages repeat administration, hence facilitating follow-ups by healthcare professionals to monitor individual’s cognitive decline (21, 22). The DCS, which takes about 6 min to administer, and is conducive regardless of assessment environment, is brief and easy to implement for large-scale screening. Moreover, although pen-and-pencil tests can allow ambiguous response from the participants to be clarified and interpreted, test results may be biased by testers with varying different experience and knowledge (23). The DCS adopts a standardized NLP system which promotes accurate analysis of verbal response from participants. It is not always feasible to recruit testers with special training or skills in case of a large-scale cognitive screening (8). Therefore, the implementation of the DCS, which can be administrated by non-professional staff or family members with no subjective bias, could lead to better management of healthcare resources by detecting cognitively impaired patients who need more comprehensive neuropsychological and clinical assessments (21).

Findings from our study showed that the DCS performed at least as well as other previous digital cognitive screening tools. For example, two recently developed digital cognitive tests which took more than 10 min to administrate showed AUCs of 0.87 and 0.91, respectively, for discriminating between demented and cognitively intact participants (10, 24) while the AUC of that in our study was 0.95. Moreover, the DCS can provide several additional advantages. First, the DCS captures participants’ spontaneous answers rather than displaying possible answer choices. It is noted that the instruments using the touchscreen to select the shown choices may cause less sensitivity and induce muscle fatigue in older adults while intuitive responses are preferred to access more reliable answers (24–26). Furthermore, many digital cognitive tests require computer literacy and higher level of functioning and thus can cause anxiety and may be not suitable for severely impaired for severely impaired individuals (21). However, the DCS features tests such as verbal recalls and semantic fluency which provide a method of intuitive answering and can be conducted among a broader range of population. Additionally, the automated scoring model make it possible to provide fast feedback without the need for a trained examiner, alleviating the related workload. As a result, from the perspectives of applicability and economics, the DCS is hopeful for a wider-range implementation in all settings, including community, primary and tertiary healthcare settings.

The voice-recognition technology enables the DSC to assess the participants’ dialogic ability which was rarely measured by previous digital cognitive tests (21). The impairment of verbal ability is predictive for the progression of cognitive impairment and incidence of dementia (27). For example, semantic fluency test requires activation of multiple cognitive processes and can explain a large amount of the diagnostic ability in a screening test (28). Therefore, whilst the DCS cannot provide substantial information in the semantic fluency test, the voice-recognition technology can be further utilized in other cognitive assessment processes to help determine impairment status. Besides that, the DCS can unobtrusively and objectively collect the changes in speech. Speech is a sensitive output system, where even slight physiological and cognitive changes may produce noticeable acoustic changes (28). Studies showed that vocal characteristics such as notably longer hesitation times and lower speech rates were detectable at the early stage of dementia (29). Therefore, speech data obtained by the DCS can inform studies focusing on differences in vocal abilities of participants responding to cognitive testing. Hence, exploration of a composite index by using the test results and acoustic parameters may further improve the DCS’s classification accuracy on different cognitive outcomes.

There are still challenges for the DCS. Firstly, the speech recognition is not sensitive to Chinese dialects at present, and it may hamper the extensive implementation of the DCS as dialects differ widely across Chinese regions, especially in rural areas with low economic level. Thus, further effort should be made to improve the DCS’s ability in recognizing Chinese dialects. Additionally, the DCS is not suitable for older adults with severe hearing or verbal impairment since they may not clearly receive the instructions or provide verbal responses on the questions. Finally, the same word list can cause learning effects considering of the longitudinal conduction of the DCS. More parallel versions of the word list will be updated based on standard verbal learning tests for future iteration.

This study has strengths. To our knowledge, this is the first digital cognitive screening tool designed for older adults in mainland China. In this study, we used 3 methods to assess the reliability of the DCS with test intervals longer than 7 days (30). Additionally, we recruited participants from both polyclinics and communities, demonstrating that our results can be generalized into primary health care and community settings.

Limitations of the present study include the small sample size and the short interval between 2 assessments in the present study has limited the interpretation on generalizability and test-retest reliability of the study results. Thus, we will further examine the utility of DCS as well as its test-retest reliability in a larger sample size with a longer retest interval. Secondly, the voice-recognition system needs more training on identifying complicated words and phrases, to improve the overall accuracy. Thirdly, a total score of 12 may have limited discrimination ability for MCI detection, as it includes less amount of test items on different cognitive domains. Further studies with a larger sample size should explore its MCI discriminant capacity by performing adjusted analysis. However, given our sample size was limited among different age and education-stratified groups, prospective adjustment of the DCS is needed to achieve more precise cut-offs at different age and education groups.

In conclusion, the DCS is a brief, reliable and valid digital dementia screening tool for older adults. The applicability of the DCS in a larger-scale community-based screening warrants further investigation.

## Data availability statement

The raw data supporting the conclusions of this article will be made available by the authors, without undue reservation.

## Ethics statement

The studies involving human participants were reviewed and approved by the Medical Ethics Committee in Zhejiang University School of Public Health (ZGL202101-1). The patients/participants provided their written informed consent to participate in this study.

## Author contributions

XZ, RH, and XX contributed to drafting the manuscript. XZ, GX, and XX contributed to performing the data analysis and interpretation of data. All authors contributed to conceptualizing the study, revising the manuscript, and approved the submitted version.

## Conflict of interest

The authors declare that the research was conducted in the absence of any commercial or financial relationships that could be construed as a potential conflict of interest.

## Publisher’s note

All claims expressed in this article are solely those of the authors and do not necessarily represent those of their affiliated organizations, or those of the publisher, the editors and the reviewers. Any product that may be evaluated in this article, or claim that may be made by its manufacturer, is not guaranteed or endorsed by the publisher.

## References

[1] Moore MJ Zhu CW Clipp EC. Informal costs of dementia care: estimates from the national longitudinal caregiver study. *J Gerontol B Psychol Sci Soc Sci.* (2001) 56:S219–28. 10.1093/geronb/56.4.s219 11445614

[2] Fitzpatrick AL Kuller LH Lopez OL Kawas CH Jagust W. Survival following dementia onset: Alzheimer’s disease and vascular dementia. *J Neurol Sci.* (2005) 229-230:43–9. 10.1016/j.jns.2004.11.022 15760618

[3] Hill NT Mowszowski L Naismith SL Chadwick VL Valenzuela M Lampit A. Computerized cognitive training in older adults with mild cognitive impairment or dementia: a systematic review and meta-analysis. *Am J Psychiatry.* (2017) 174:329–40. 10.1176/appi.ajp.2016.16030360 27838936

[4] Roberts R Knopman DS. Classification and epidemiology of MCI. *Clin Geriatr Med.* (2013) 29:753–72. 10.1016/j.cger.2013.07.003 24094295 PMC3821397

[5] Dubois B Padovani A Scheltens P Rossi A Dell’Agnello G. Timely diagnosis for Alzheimer’s disease: a literature review on benefits and challenges. *J Alzheimers Dis.* (2016) 49:617–31. 10.3233/JAD-150692 26484931 PMC4927869

[6] Tsoi KK Chan JY Hirai HW Wong SY Kwok TC. Cognitive tests to detect dementia: a systematic review and meta-analysis. *JAMA Intern Med.* (2015) 175:1450–8. 10.1001/jamainternmed.2015.2152 26052687

[7] Chan J Kwong J Wong A Kwok T Tsoi K. Comparison of computerized and paper-and-pencil memory tests in detection of mild cognitive impairment and dementia: a systematic review and meta-analysis of diagnostic studies. *J Am Med Dir Assoc.* (2018) 19:748–56. 10.1016/j.jamda.2018.05.010 29921507

[8] Koo BM Vizer LM. Mobile technology for cognitive assessment of older adults: a scoping review. *Innov Aging.* (2019) 3:igy038. 10.1093/geroni/igy038 30619948 PMC6312550

[9] Webb SS Kontou E Demeyere N. The COVID-19 pandemic altered the modality, but not the frequency, of formal cognitive assessment. *Disabil Rehabil.* (2021) 1–9. 10.1080/09638288.2021.1963855 34397311

[10] Bloniecki V Hagman G Ryden M Kivipelto M. Digital screening for cognitive impairment - a proof of concept study. *J Prev Alzheimers Dis.* (2021) 8:127–34. 10.14283/jpad.2021.2 33569558

[11] Inoue M Jimbo D Taniguchi M Urakami K. Touch panel-type dementia assessment scale: a new computer-based rating scale for Alzheimer’s disease. *Psychogeriatrics.* (2011) 11:28–33. 10.1111/j.1479-8301.2010.00345.x 21447106

[12] Suzumura S Osawa A Maeda N Sano Y Kandori A Mizuguchi T Differences among patients with Alzheimer’s disease, older adults with mild cognitive impairment and healthy older adults in finger dexterity. *Geriatr Gerontol Int.* (2018) 18:907–14. 10.1111/ggi.13277 29512255

[13] Tung JY Rose RV Gammada E Lam I Roy EA Black SE Measuring life space in older adults with Mild-to-Moderate Alzheimer’s disease using mobile phone GPS. *Gerontology.* (2014) 60:154–62. 10.1159/000355669 24356464

[14] Pendlebury ST Welch SJV Cuthbertson FC Mariz J Mehta Z Rothwell PM. Telephone assessment of cognition after transient ischemic attack and stroke modified telephone interview of cognitive status and telephone montreal cognitive assessment versus face-to-face montreal cognitive assessment and neuropsychological battery. *Stroke.* (2013) 44:227–367. 10.1161/STROKEAHA.112.673384 23138443 PMC5593099

[15] Zietemann V Kopczak A Muller C Wollenweber FA Dichgans M. Validation of the telephone interview of cognitive status and telephone Montreal cognitive assessment against detailed cognitive testing and clinical diagnosis of mild cognitive impairment after stroke. *Stroke.* (2017) 48:2952–7. 10.1161/STROKEAHA.117.017519 29042492

[16] Dong Y Lee WY Hilal S Saini M Wong TY Chen CL Comparison of the Montreal cognitive assessment and the mini-mental state examination in detecting multi-domain mild cognitive impairment in a Chinese sub-sample drawn from a population-based study. *Int Psychogeriatr.* (2013) 25:1831–8. 10.1017/S1041610213001129 23870281

[17] Ng TP Feng L Lim WS Chong MS Lee TS Yap KB Montreal cognitive assessment for screening mild cognitive impairment: variations in test performance and scores by education in Singapore. *Dement Geriatr Cogn Disord.* (2015) 39:176–85. 10.1159/000368827 25572449

[18] Zhang Z Takanobu R Zhu Q Huang M Zhu X. Recent advances and challenges in task-oriented dialog systems. *Sci China Technol Sci.* (2020) 63:2011–27. 10.1007/s11431-020-1692-3

[19] Forbes-McKay KE Venneri A. Detecting subtle spontaneous language decline in early Alzheimer’s disease with a picture description task. *Neurol Sci.* (2005) 26:243–54. 10.1007/s10072-005-0467-9 16193251

[20] Pinto T Machado L Bulgacov TM Rodrigues-Junior AL Costa M Ximenes R Influence of age and education on the performance of elderly in the Brazilian version of the Montreal cognitive assessment battery. *Dement Geriatr Cogn Disord.* (2018) 45:290–9. 10.1159/000489774 29996142

[21] Lee JY Dong WL Cho SJ Na DL Hong JJ Kim SK Brief screening for mild cognitive impairment in elderly outpatient clinic: validation of the Korean version of the Montreal cognitive assessment. *J Geriatr Psychiatry Neurol.* (2008) 21:104–10. 10.1177/0891988708316855 18474719

[22] Zygouris S Tsolaki M. Computerized cognitive testing for older adults: a review. *Am J Alzheimers Dis Other Demen.* (2015) 30:13–28. 10.1177/1533317514522852 24526761 PMC10852880

[23] Darby DG Pietrzak RH Fredrickson J Woodward M Moore L Fredrickson A Intraindividual cognitive decline using a brief computerized cognitive screening test. *Alzheimers Dement.* (2012) 8:95–104. 10.1016/j.jalz.2010.12.009 22404851

[24] Chan J Wong A Yiu B Mok H Lam P Kwan P Electronic cognitive screen technology for screening older adults with dementia and mild cognitive impairment in a community setting: development and validation study (vol 22, e17332, 2020). *J Med Internet Res.* (2021) 23:e26724. 10.2196/26724 33337341 PMC7775823

[25] Inoue M Jinbo D Nakamura Y Taniguchi M Urakami K. Development and evaluation of a computerized test battery for Alzheimer’s disease screening in community-based settings. *Am J Alzheimers Dis Other Demen.* (2009) 24:129–35. 10.1177/1533317508330222 19150968 PMC10846275

[26] Tierney MC Lermer MA. Computerized cognitive assessment in primary care to identify patients with suspected cognitive impairment. *J Alzheimers Dis.* (2010) 20:823–32. 10.3233/JAD-2010-091672 20413868

[27] McDonnell M Dill L Panos S Amano S Brown W Giurgius S Verbal fluency as a screening tool for mild cognitive impairment. *Int Psychogeriatr.* (2020) 32:1055–62. 10.1017/S1041610219000644 31258101 PMC9153280

[28] Sutin AR Stephan Y Terracciano A. Verbal fluency and risk of dementia. *Int J Geriatr Psychiatry.* (2019) 34:863–7. 10.1002/gps.5081 30729575 PMC6530594

[29] Konig A Satt A Sorin A Hoory R Derreumaux A David R Use of speech analyses within a mobile application for the assessment of cognitive impairment in elderly people. *Curr Alzheimer Res.* (2018) 15:120–9. 10.2174/1567205014666170829111942 28847279

[30] Park MS Kang KJ Jang SJ Lee JY Chang SJ. Evaluating test-retest reliability in patient-reported outcome measures for older people: a systematic review. *Int J Nurs Stud.* (2018) 79:58–69. 10.1016/j.ijnurstu.2017.11.003 29178977

